# Carbon Dots and Their Functionalization with Photosensitizer Chlorin E6: Advancing Antibacterial Efficacy Through Enhanced Photodynamic Effects

**DOI:** 10.3390/pharmaceutics18040487

**Published:** 2026-04-15

**Authors:** Siqi Wang, Colin P. McCoy, Peifeng Li, Yining Li, Yinghan Zhao, Gavin P. Andrews, Yi Ge

**Affiliations:** School of Pharmacy, Queen’s University Belfast, 97 Lisburn Road, Belfast BT9 7BL, UK; swang54@qub.ac.uk (S.W.); c.mccoy@qub.ac.uk (C.P.M.); pli07@qub.ac.uk (P.L.); yli80@qub.ac.uk (Y.L.); yzhao32@qub.ac.uk (Y.Z.); g.andrews@qub.ac.uk (G.P.A.)

**Keywords:** microwave-assisted synthesis, carbon dots, photosensitizer, antibacterial efficacy, photodynamic effects

## Abstract

**Background/Objectives**: Carbon dots (CDs) are promising antimicrobial nanomaterials owing to their biocompatibility, environmental friendliness, and tunable surface chemistry. This study aimed to synthesize nitrogen-doped CDs (AS-CDs) and develop a light-responsive antibacterial system through conjugation with chlorin e6 (Ce6). **Methods**: AS-CDs were synthesized by a microwave-assisted method using L-ascorbic acid and spermidine, followed by conjugation with Ce6. The materials were characterized by transmission electron microscopy, zeta potential analysis, and spectroscopic methods, and their antibacterial activity was evaluated against *Escherichia coli*, *Staphylococcus aureus*, and methicillin-resistant *S. aureus* (MRSA) under both dark and visible-light conditions. Cytotoxicity was assessed using HaCaT cells. **Results**: The AS-CDs exhibited a uniform nanoscale morphology with an average diameter of 6.3 nm and a positive surface charge of +15.6 mV, together with intrinsic broad-spectrum antibacterial activity. Ce6 conjugation further enhanced antibacterial efficacy under light irradiation, with the CDs-Ce6 conjugate achieving complete eradication of *S. aureus* and MRSA and marked inhibition of *E. coli* at 2.5 μg/mL. Cytotoxicity studies demonstrated low toxicity in HaCaT cells within the effective antibacterial concentration range. **Conclusions**: These findings highlight the potential of microwave-synthesized, photosensitizer-conjugated CDs as next-generation antimicrobial agents. This platform offers a cost-effective, sustainable, eco-friendly, and efficient platform for combating bacterial infections, with broader potential in pharmaceutical and biomedical applications.

## 1. Introduction

Antimicrobial resistance (AMR) continues to pose a significant global health challenge, diminishing the efficacy of conventional antibiotics and complicating the treatment of bacterial infections. Addressing this issue requires the development of innovative approaches that move beyond traditional therapies. Among various emerging strategies, carbon dots (CDs) have attracted growing interest as potential nanomaterials for combating bacterial infections due to their unique properties, such as high biocompatibility, environmental sustainability, and ease of surface modification [1,2,3]. Recent studies have further demonstrated that CDs can be engineered for synergistic antibacterial effects by integrating multiple functionalities, such as antibiotic-derived precursors [4], photosensitizers, or photothermal agents [5].

CDs, which are small, carbon-based nanoparticles, exhibit remarkable optical properties, including tunable photoluminescence and the ability to generate reactive oxygen species (ROS) [6,7], and the ability to interact with bacterial membranes and biofilms. These features make them promising candidates for antimicrobial photodynamic therapy (PDT) and photothermal therapy (PTT), where light or heat triggers ROS production or localized thermal stress, enhancing bacterial killing [5,8,9,10]. However, while CDs show promise, their intrinsic antibacterial effects are often limited, particularly against resistant strains such as *Escherichia coli* and methicillin-resistant *Staphylococcus aureus* (MRSA) [11,12]. Surface functionalization—including heteroatom doping, conjugation with photosensitizers, or attachment of functional moieties—can further improve antibacterial performance, facilitate biofilm penetration, and target multidrug-resistant bacteria [13,14,15].

In addition, the physicochemical properties and antibacterial performance of carbon dots are highly dependent on their synthesis methods, including precursor selection, reaction conditions, and doping strategies [6]. Microwave-assisted synthesis has emerged as an efficient and sustainable technique for producing CDs with precise control over particle size and surface properties [16,17]. This approach not only shortens reaction times but also allows for the homogeneous heating of precursors, leading to consistent and high-quality nanomaterials. Furthermore, doping CDs with nitrogen, achieved by introducing precursors like spermidine during synthesis, can enhance their photophysical properties and ROS-generation capability, making them more effective in biomedical applications [18,19,20,21,22,23]. Pairing nitrogen-doped CDs with photosensitizers, such as Chlorin e6 (Ce6), holds significant promise. Ce6 is known for its strong absorption of red and near-infrared light, high ROS-generating efficiency, and potential for deeper tissue penetration, making it an excellent candidate for PDT [24,25].

Despite these advancements, there remains a gap in understanding how nitrogen-doped CDs functionalized with Ce6 can be harnessed for enhanced light-activated antibacterial applications. Key questions include whether such conjugates can significantly improve antibacterial efficacy against diverse bacterial strains and whether they can maintain low toxicity for safe clinical use.

**Scheme 1:**
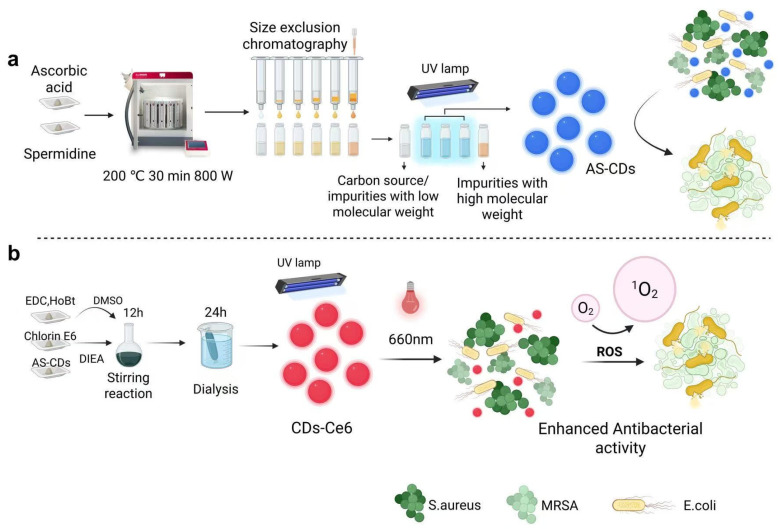
(**a**) Synthesis of AS-CDs via microwave-assisted approach and antibacterial activity of AS-CDs. (**b**) Synthesis of CDs-Ce6 conjugate and bactericidal activities of CDs-Ce6 upon 660 nm illumination.

This study seeks to address these gaps by synthesizing nitrogen-doped CDs using an advanced microwave-assisted method with L-ascorbic acid and spermidine as precursors (Scheme 1a). The CDs are further functionalized with Ce6 to create a versatile CDs-Ce6 conjugate (Scheme 1b). The antibacterial properties of these nanomaterials, both under light exposure and in the absence of light, are evaluated against *E. coli*, *S. aureus*, and MRSA. Notably, CDs-Ce6 significantly enhanced its antibacterial efficacy under light irradiation conditions. Cytotoxicity studies are also conducted to assess their potential biomedical applicability. The findings aim to provide a foundation for developing advanced carbon-based nanomaterials capable of tackling AMR through enhanced photodynamic mechanisms.

## 2. Materials and Methods

### 2.1. Synthesis of AS-CDs and A-CDs

A 0.5 g mixture of L-ascorbic acid and spermidine tetrahydrochloride (1:2 molar ratio) was dissolved in 10 mL of DI water and transferred to a SK-15eT vessel (Milestone Srl, Sorisole, Italy). The sealed vessel was heated at 200 °C for 30 min using a Milestone FlexiWAVE microwave reactor (Milestone Srl, Sorisole, Italy) operating at 800 W. After cooling to room temperature, the supernatant was collected and purified using Sephadex^®^ G-10 (Cytiva, Uppsala, Sweden) gel permeation chromatography. The purified product was freeze-dried for 24 h and stored at 4 °C. For the control sample (A-CDs), 0.5 g of L-ascorbic acid was dissolved in 10 mL of DI water, and the solution was processed under identical microwave conditions at 180 °C. Purification and storage methods were the same as those used for AS-CDs. The synthesis was repeated ≥3 times to ensure consistency of physicochemical properties and reproducibility of the experiment.

### 2.2. Synthesis of CDs-Ce6 Conjugate

The AS-CDs were conjugated with Ce6 using EDC/HoBt-mediated coupling chemistry (Scheme 1b). Ce6 (0.5 mg/mL) was first activated by dissolving it in anhydrous DMF and adding equimolar amounts of HoBt and 1.1 equivalents of EDC, followed by stabilization with 1.1 equivalents of diisopropylethylamine (DIEA). This solution was stirred for 30 min under dark conditions. AS-CDs (0.5 mg/mL) were then added to the activated Ce6 solution at a volume ratio of 2:1 and stirred for 12 h at room temperature in the dark. The resulting mixture was dialyzed against DI water for 24 h using a 3.5 kDa molecular weight cut-off dialysis membrane to remove unreacted reagents and byproducts. The purified CDs-Ce6 conjugate was freeze-dried for 24 h and stored at 4 °C. The synthesis was repeated ≥3 times to ensure consistency of physicochemical properties and reproducibility of the experiment.

### 2.3. Characterization of CDs and CDs-Ce6 Conjugate

The morphology of AS-CDs and CDs-Ce6 conjugates was analyzed using transmission electron microscopy (TEM) on a JEOL JEM-1400 Flash Electron Microscope (JEOL Ltd., Tokyo, Japan). Particle size distribution and zeta potential measurements were conducted using dynamic light scattering (Nano ZS90 System, Malvern Instruments Ltd., Malvern, UK). The optical properties were studied using UV-visible spectroscopy (Cary 60 UV–Vis spectrophotometer, Agilent Technologies LDA UK Ltd., Stockport, UK), fluorescence spectroscopy (F-2710 fluorescence spectrophotometer, Hitachi High-Tech Europe GmbH., Maidenhead, UK), and Fourier transform infrared (FT-IR) spectroscopy (Spectrum Two N spectrometer, PerkinElmer, Shelton, CT, USA). The crystallinity and structure were examined via X-ray powder diffraction (XRD) using a D8 Advance Eco X-ray Diffractometer (Bruker UK Ltd., Coventry, UK).

### 2.4. Bacterial Culture

Stored bacterial strains (*S. aureus*, *E. coli*, and MRSA) were retrieved from a −80 °C freezer and thawed in a water bath at 37 °C for 3 min. Using a sterile inoculation loop, each strain was transferred into a flask containing Mueller Hinton Broth (MHB). The cultures were incubated in an orbital shaker at 37 °C and 100 rpm. The overnight cultures were then subcultured (1:100) into fresh MHB and incubated for approximately 4 h to reach the mid-logarithmic growth phase. Cells were harvested by centrifugation at 3000 rpm for 12 min and resuspended in fresh medium to an optical density of OD_600_ = 0.3. The bacterial suspension was further diluted 1:100 to obtain a working inoculum of approximately 1 × 10^6^ CFU/mL for subsequent antibacterial assays.

### 2.5. Agar Plate Well Diffusion Test

The antibacterial activity of AS-CDs was initially assessed using the agar well diffusion method. Agar plates were inoculated with 50 µL of microbial suspension (10^6^ CFU/mL) and evenly spread over the entire surface. Four wells, each 1 cm in diameter, were created on the agar surface using a sterile tip. AS-CDs solutions at varying concentrations (20, 10, 5, 2.5, and 1.25 mg/mL) were prepared in deionized water, sterilized using a 0.22 µm membrane filter, and 100 µL of each solution was added to one well per plate. As controls, 100 µL of MHB, 100 µL of a carbon source solution, and 100 µL of A-CDs solution (20 mg/mL) were added to the remaining wells. The plates were incubated overnight at 37 °C, and the zone of inhibition was measured to evaluate antibacterial efficacy.

### 2.6. Minimum Inhibitory Concentration (MIC) and Minimum Bactericidal Concentration (MBC) Determination

The MIC of AS-CDs was determined using a broth dilution method. Suspensions of *S. aureus*, *E. coli*, and MRSA (1 × 10^6^ CFU/mL) were treated with AS-CDs at various final concentrations (10, 5, 2.5, 1.25, 0.625, 0.3125, 0.1563, 0.0781, 0.0391, and 0.0195 mg/mL) in a 96-well plate. Each well contained 100 µL of bacterial suspension and 100 µL of MHB. A control well with MHB and bacterial suspension, but without AS-CDs, was included to confirm bacterial viability and serve as a baseline. After 24 h of incubation at 37 °C, bacterial growth was visually assessed. The MIC was defined as the lowest concentration of AS-CDs at which no visible bacterial growth occurred.

For the MBC, aliquots from wells with no visible growth were streaked onto agar plates and incubated further overnight. The MBC was determined as the lowest concentration of AS-CDs that resulted in no bacterial colonies, indicating bactericidal activity.

### 2.7. Kinetic Curves

Bacterial suspensions of *S. aureus*, *E. coli*, and MRSA were cultured in MHB to the mid-logarithmic growth phase, achieving a final concentration of 10^6^ CFU/mL. The suspensions were then treated with AS-CDs at varying concentrations (2.5, 1.25, 0.625, and 0.313 mg/mL) and incubated at 37 °C. Optical density at 600 nm (OD_600_) was measured at 0, 2, 4, 6, 8, 10, 12, 14, 16, 18, 20, 22, and 24 h to monitor bacterial growth. Growth curves, describing the changes in OD_600_ over time, were generated using a microplate reader (Thermo Scientific Varioskan LUX, Warrington, UK) with 96-well plates.

### 2.8. Antibacterial Activity Evaluation with Photoexcitation

Dilutions of AS-CDs were prepared in PBS to achieve final concentrations of 10, 5, 2.5, 1.25, 0.625, 0.313, 0.16, 0.08, 0.04, 0.02, and 0.01 mg/mL. CDs-Ce6 solutions were prepared at concentrations of 5, 2.5, 1.25, 0.625, and 0.313 µg/mL. Chlorin e6 (Ce6) was also diluted to the same concentrations to serve as a control group. For each treatment, 100 µL of the AS-CDs, CDs-Ce6, or Ce6 solutions was added to designated wells of a 96-well plate, along with 100 µL of bacterial suspension (10^6^ CFU/mL).

The mixtures were either exposed to LED light (660 nm, 284 mW/cm^2^) for 30 min or kept in the dark. The following controls were included on each plate: (a) growth control: bacteria cultured in MHB without any treatment; (b) light-only control: bacteria with light exposure but without CDs; (c) dark control: bacteria with CDs but without light exposure. After light treatment, the plates were incubated at 37 °C for 24 h to allow for bacterial growth. The OD_600_ of each well was measured using a spectrophotometer to assess bacterial growth, with lower optical density values indicating stronger antibacterial activity. The antibacterial activity of photo-activated AS-CDs and CDs-Ce6 was quantified by measuring relative bacterial viability, which was defined as the ratio of the optical density of the treated group to that of the untreated control (without AS-CDs and CDs-Ce6 treatment).

To further evaluate bacterial viability, 10 µL of the bacterial suspension from each well in the treatment groups was spread onto agar plates after the 24 h incubation. The agar plates were then incubated overnight at 37 °C to observe bacterial growth.

### 2.9. Intracellular ROS Detection

Intracellular ROS generation was detected using the fluorescent probe 2′,7′-dichlorodihydrofluorescein diacetate (DCFDA) [26]. DCFDA readily crosses cell membranes and serves as a substrate for ROS. Once inside the cell, intracellular esterases cleave the acetate groups from DCFDA, converting it into the non-fluorescent compound 2′,7′-dichlorodihydrofluorescein (DCFH). In the presence of ROS, DCFH is rapidly oxidized to 2′,7′-dichlorofluorescein (DCF), a highly fluorescent compound. The fluorescence intensity of DCF is directly proportional to the ROS levels within the cells.

For this study, *S. aureus*, *E. coli*, and MRSA were cultured in appropriate broth at 37 °C for 18 h. The bacterial suspensions were centrifuged at 3000× *g* for 10 min, and the pellets were resuspended to an optical density of OD_600_ = 0.3, corresponding to approximately 1 × 10^8^ CFU/mL. 10 mM DCFDA stock solution was prepared in DMSO, protected from light, and stored at −20 °C. Immediately before use, the stock solution was diluted in PBS to a final concentration of 20 µM.

For each well of a 96-well plate, 100 µL of the DCFDA solution and 100 µL of bacterial suspension were added, resulting in a total volume of 200 µL. The plates were incubated at 37 °C in the dark for 30 min to allow DCFDA to penetrate the cells and be hydrolyzed by intracellular esterases, forming DCFH. After incubation, the mixtures were centrifuged at 10,000× *g* for 10 min, and the supernatants were removed. The bacterial pellets were resuspended in the appropriate sample solutions (AS-CDs, Ce6, and CDs-Ce6) and subjected to light treatment for 30 min. The mixtures were then incubated in the dark at 37 °C for an additional 2 h to allow DCFH oxidation to DCF.

Fluorescence intensity was measured at an excitation wavelength of 488 nm using a fluorescence spectrophotometer. The increase in fluorescence intensity compared to control samples indicated the level of ROS production within the bacterial cells.

### 2.10. Cellular Toxicity Test

The cytotoxicity of AS-CDs and CDs-Ce6 conjugates was assessed using HaCaT cells (human keratinocyte cell line), which were obtained from Caltag Medsystems Ltd. (Buckingham, UK) (cellosaurus accession number CVCL_0038). HaCaT cells were seeded at a density of 1 × 10^5^ cells/mL in a 96-well culture plate (100 µL per well) with complete growth medium (CGM) containing Dulbecco’s Modified Eagle’s Medium (DMEM) supplemented with 10% fetal bovine serum (FBS). The cells were incubated for 24 h at 37 °C in a humidified atmosphere with 5% CO_2_. After incubation, the medium was replaced with 100 µL of varying concentrations of N-doped CDs dissolved in CGM. Following a 24 h exposure, the medium was removed, and the cells were washed three times with PBS. A mixture of 10 µL MTT reagent and 90 µL DMEM was then added to each well, and the plate was incubated for 1 h. The medium was carefully discarded, and 150 µL of DMSO was added to dissolve the formazan crystals. The optical density (OD) was measured at 538 nm to quantify cell viability. The following controls were included on each plate: (a) growth control: cells cultured in CGM without test compounds; (b) positive control: cells treated with 0.1% Triton X-100 in CGM; (c) blank control: wells containing CGM only, without cells (background correction). Sample-only blanks (CGM + CDs + MTT, no cells) were included where needed to correct for optical interference.

The cell viability was estimated using the following equation:

(1) $$Cell\;viability\;\left[\%\right]=\left({OD}_{treated}-{OD}_{blank}/{OD}_{growth\;control}-{OD}_{blank}\right)\times 100\%$$

where *OD_treated_* and *OD_growth control_* were the optical density of cells in the presence and absence of CDs, respectively.

### 2.11. Statistical Analysis Method

In this study, statistical analyses were performed to assess significant differences between experimental groups. Data are presented as mean ± standard deviation (SD) from at least three independent experiments. For comparisons involving two groups, an independent samples *t*-test was used to determine statistical significance in the means. For comparisons involving more than two groups, a one-way analysis of variance (ANOVA) was conducted to identify significant differences. Post hoc analyses, including Tukey’s Honestly Significant Difference (HSD) test, were applied to pinpoint specific group differences. A *p*-value of less than 0.05 was considered statistically significant. All statistical analyses were conducted using GraphPad Prism version 8.4.3.

## 3. Results and Discussion

### 3.1. Synthesis and Characterization of AS-CDs

**Figure 1:**
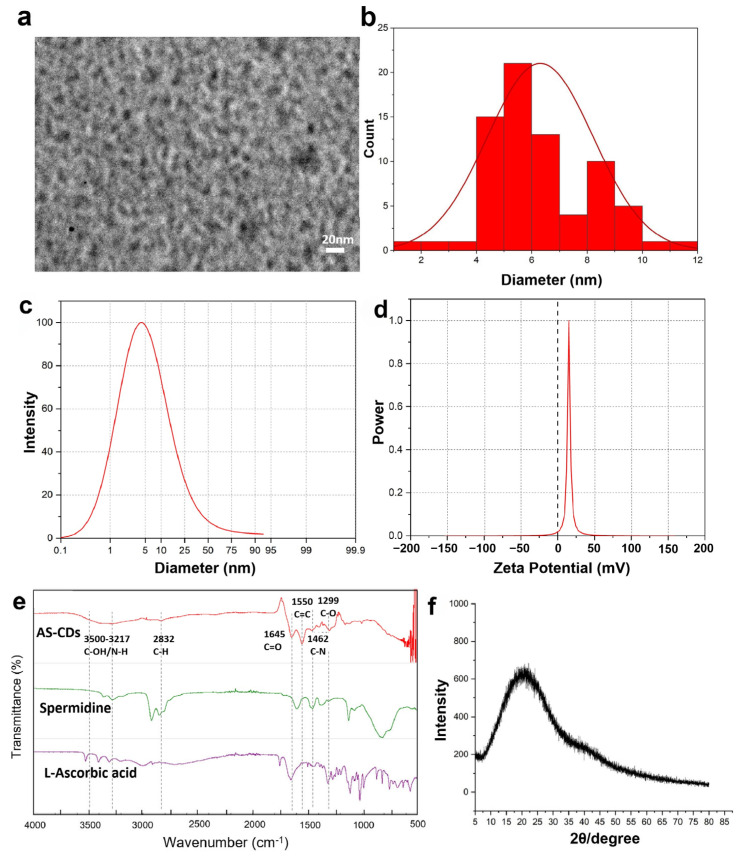
Characterization of ASCDs. (**a**) TEM analysis of AS-CDs. (**b**) Size distribution of AS-CDs. (**c**) Particle size distribution of AS-CDs by DLS. (**d**) Zeta potential analysis of AS-CDs. (**e**) FTIR spectra of AS-CDs, L-ascorbic acid and spermidine. (**f**) XRD analysis of AS-CDs.

The TEM image (Figure 1a) revealed that the synthesized AS-CDs were spherical and uniformly distributed, with particle sizes ranging from 4.0 to 10.5 nm and an average diameter of 6.3 nm (Figure 1b). A small fraction of the AS-CDs exhibited slight aggregation, forming irregular clusters. This behavior was likely due to the high surface energy and the presence of abundant functional groups (e.g., –OH, –NH_2_, –COOH) on the CD surfaces, which can promote intermolecular interactions such as hydrogen bonding and van der Waals forces. Additionally, the drying process during TEM sample preparation may further facilitate particle clustering. Despite this minor aggregation, the overall nanoscale size and spherical morphology of the AS-CDs remain well-preserved.

Dynamic light scattering (DLS) confirmed this size distribution and measured a positive zeta potential of +15.6 mV (Figure 1c,d), attributed to the amino groups derived from spermidine. This positive charge is beneficial for interacting with the negatively charged bacterial cell membranes, enhancing antibacterial activity.

Fourier transform infrared (FT-IR) spectroscopy was used to analyze the functional groups present on the surface of AS-CDs. As shown in Figure 1e, the broad band at 3496–3317 cm^−1^ corresponded to the stretching vibrations of O–H and N–H groups. The peak at 2956 cm^−1^ was attributed to the stretching vibrations of C–H bonds. A strong peak observed at 1650 cm^−1^ indicated the stretching vibration of the C=O bond in amide groups, suggesting an amidation reaction between the carboxylic acids in L-ascorbic acid and the amines in spermidine. Additionally, the peak at 1556 cm^−1^ corresponded to the bending vibration of C=C bonds. Peaks at 1365 cm^−1^ and 1192 cm^−1^, attributed to the stretching vibrations of C–N and C–O bonds, respectively, confirmed the presence of nitrogen atoms and carbonyl groups in AS-CDs. Comparison with the spectra of L-ascorbic acid and spermidine further supported the successful synthesis of the new product, as evidenced by the distinct presence of C=O and C=C bonds in the AS-CDs.

The presence of AS-CDs was further confirmed using X-ray diffraction (XRD) analysis (Figure 1f). The XRD pattern exhibited a broad single peak, characteristic of the amorphous or semi-crystalline nature of the material. This broad peak, occurring between 2θ of 20° and 30°, was indicative of graphitic carbon structures. The specific diffraction angle of AS-CDs was measured at 22.18°, corresponding to an interlayer lattice spacing of 0.40 nm. This spacing was slightly larger than that of graphene layers (0.335 nm), likely due to structural defects and the incorporation of functional groups such as hydroxyl and amino groups.

**Figure 2:**
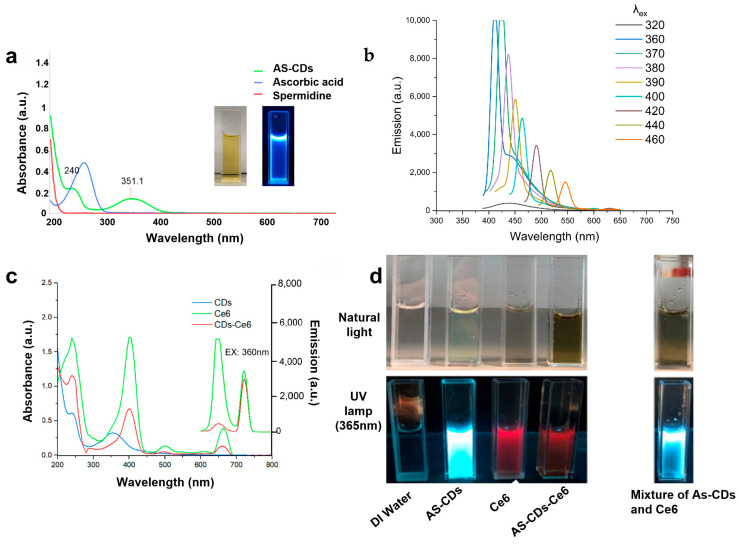
Characterization of AS-CDs and CDs-Ce6. (**a**) UV-vis absorption spectra of AS-CDs (0.1 mg/mL), ascorbic acid (0.1 mg/mL) and spermidine (0.1 mg/mL), with inserted photographs of AS-CDs under daylight (left) and a UV lamp (365 nm, right). (**b**) Fluorescence emission spectra of AS-CDs (0.25 μg/mL) under varied excitation wavelengths. (**c**) UV−vis absorption and fluorescence (excitation 360 nm) spectra of AS-CDs and CDs-Ce6. The absorption spectrum of AS-CDs, CDs-Ce6 and Ce6 were measured at 0.25 mg/mL, and the fluorescence emission spectrum of CDs-Ce6 and Ce6 were recorded at 0.25 µg/mL. (**d**) Photographs of CDs-Ce6 conjugate and source solutions under UV light (365 nm). From left to right: DI water; AS-CDs dissolved in DI water; Ce6 dissolved in DMSO; CDs-Ce6 dissolved in DI water; Mixture of AS-CDs and Ce6 in DI water.

The optical properties of AS-CDs were analyzed using UV-visible and fluorescence spectroscopy. In the UV-visible spectrum of AS-CDs (Figure 2a), an absorption peak at 240 nm was attributed to π→π* transitions (C=C bonds), while a secondary peak at 351.1 nm corresponded to n→π* transitions (C=O or C=N bonds). Compared to L-ascorbic acid, which exhibited a single absorption peak at 260.4 nm, and spermidine, which showed no absorption in the UV region, these distinct peaks confirm the formation of a new product. Additionally, the reaction solution changed from transparent to yellow, and the resulting AS-CDs displayed strong blue fluorescence under UV light (356 nm), further verifying their successful synthesis.

Fluorescence emission spectra revealed that the photoluminescence of AS-CDs was strongly dependent on the excitation wavelength (Figure 2b). As the excitation wavelength increased from 320 nm to 460 nm, the maximum emission wavelength exhibited a red shift, ranging from 400 nm to 550 nm. This excitation-wavelength-dependent behavior suggested the presence of multiple emissive states or a broad size distribution within the AS-CDs. These variations allowed AS-CDs to absorb and emit light at different wavelengths, with changes in emission intensity influenced by the efficiency of each emissive state or size population under different excitation conditions.

### 3.2. Synthesis and Characterization of CDs-Ce6 Conjugate

The synthesized CDs-Ce6 conjugate was characterized using UV-Vis, IR, and fluorescence (FL) spectroscopy, revealing distinctive spectral features consistent with the successful incorporation of Ce6 into the carbon dot system (Figure 2c,d). The UV-Vis absorption spectrum displayed a peak at 240 nm, corresponding to the π→π* electron transition of C=C bonds, which was also observed for AS-CDs and suggested the retention of the carbon-dot-related structural features. Additional peaks at 400 nm (Soret band), 500 nm, and 660 nm (Q bands) were observed, which were associated with the porphyrin structure of chlorin e6 and were consistent with previously reported carbon dot–chlorin e6 systems by Beack [27]. The FL emission spectrum exhibited a significant red shift from 410 nm for AS-CDs to 650 nm for CDs-Ce6, which was consistent with the contribution of the porphyrin moiety and supported the successful functionalization of AS-CDs with Ce6.

As shown in Figure 2d, the solution’s color remained unchanged under natural light but transitioned from blue to a strong red fluorescence under UV light (365 nm). Additionally, CDs-Ce6 exhibited improved aqueous dispersibility compared with free Ce6, which was poorly soluble in aqueous media. Notably, the fluorescence color of the mixture of AS-CDs and Ce6 was blue, further suggesting that the optical behavior of the obtained product differed from that of the unreacted components.

**Figure 3:**
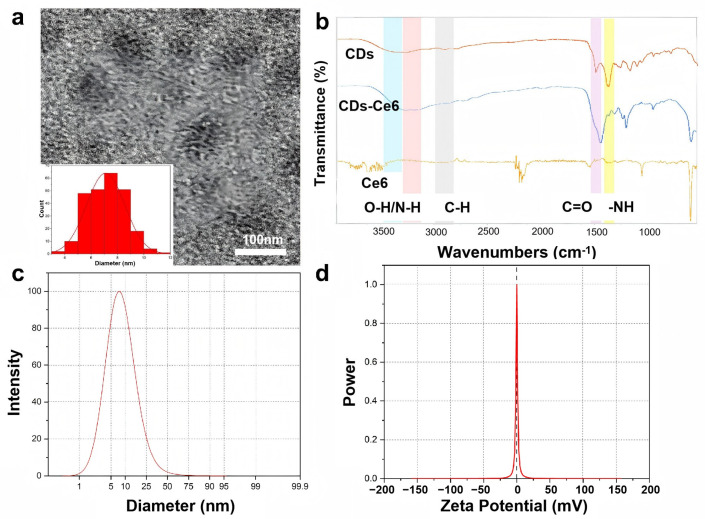
Characterization of CDs-Ce6. (**a**) TEM image showing the morphology of CDs-Ce6 conjugates and particle size distribution derived from TEM analysis (insert). (**b**) FTIR spectra of AS-CDs and CDs-Ce6. (**c**) Particle size distribution of CDs-Ce6 by DLS. (**d**) Zeta potential analysis of CDs-Ce6.

IR spectroscopy also provided supportive evidence for the formation of the CDs-Ce6 system, showing characteristic bands assigned to C-OH/NH (3500–3150 cm^−1^), C-H (3000–2800 cm^−1^), C=O (1650 cm^−1^), and -NH (1365 cm^−1^) (Figure 3b). Compared with the precursor materials, the retention of these characteristic absorption features together with changes in band intensity and spectral profile suggested that functional groups associated with both AS-CDs and Ce6 were present in the final purified product and experienced an altered local chemical environment after reaction. Taken together, these results supported the successful formation of a Ce6-functionalized carbon dot system, in which the porphyrin structure contributed to the altered optical and functional properties.

TEM analysis showed that the obtained CDs-Ce6 exhibited a relatively uniform and spherical morphology (Figure 3a). Image analysis was performed using Fiji (ImageJ 1.54f, National Institutes of Health, Bethesda, MD, USA), which gave an average particle diameter of 6.107 ± 1.452 nm, which was in agreement with the size distribution measured by dynamic light scattering (DLS) (Figure 3c). This size was larger than that of AS-CDs, which may be attributed to the introduction of Ce6 onto the carbon dot surface.

In addition, zeta potential measurements via DLS indicated that the CDs-Ce6 product had a near-neutral surface charge (Figure 3d). This change may reflect the altered surface characteristics after Ce6 functionalization. Despite the surface charge approaching zero, the CDs-Ce6 conjugates maintain a uniform particle distribution. This apparent stability can be attributed to additional non-electrostatic mechanisms, such as steric hindrance from the surface-bound Ce6 molecules and solvation effects in the aqueous medium, which prevent close particle–particle contact and contribute to maintaining uniform dispersion [28,29,30].

### 3.3. Antibacterial Activity of AS-CDs Without Photoexcitation

The antimicrobial effect of synthesized AS-CDs against *S. aureus*, *E. coli*, and MRSA was preliminarily evaluated using the agar-plate diffusion method. For comparison, antimicrobial activities of the carbon source solution (20 mg/mL) and A-CDs (synthesized solely from L-ascorbic acid) were also assessed.

**Figure 4:**
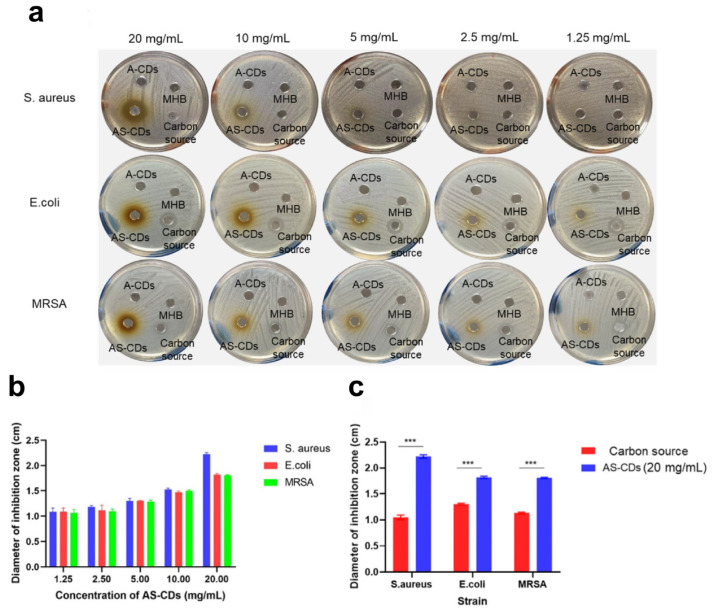
Antibacterial activity of AS-CDs against *S. aureus*, MRSA, and *E. coli*. evaluated by agar-plate well diffusion method. (**a**) Representative images of inhibition zones for the three bacterial strains treated with AS-CDs at various concentrations (1.25–20 mg/mL). The indicated Gram-negative bacteria were seeded on the agar media, and holes were made with a cork-borer (1 cm in diameter). The bottom of the holes was sealed with 0.5% agar, and 100 µL of CDs (1.25–20 mg/mL) were added to each well. (**b**) Quantitative measurement of inhibition zone diameters. (**c**) Comparison of antibacterial effects between AS-CDs (20 mg/mL) and the carbon source solution. Values were mean (from three replicates) ± standard deviation. One-way ANOVA, *** *p* = 0.001.

Figure 4a,b illustrate the inhibition zones and their sizes for the three bacterial strains treated with varying concentrations of AS-CDs. Neither the A-CDs nor the MHB control group (without sample) produced any inhibition zones. AS-CDs began exhibiting antibacterial activity at a concentration of 2.5 mg/mL, with consistent inhibition observed across all three strains. As the concentration of AS-CDs increased, the inhibition zones expanded significantly for all bacteria tested. While the carbon source solution displayed some growth inhibition, AS-CDs demonstrated significantly larger inhibition zones, particularly at the highest concentration (20 mg/mL). At this concentration, AS-CDs showed stronger antibacterial effects against *S. aureus* and *E. coli* compared to the carbon source, as seen in Figure 4c. These findings confirmed that AS-CDs possess potent antibacterial properties against both Gram-positive and Gram-negative bacteria, highlighting their potential as effective antimicrobial agents.

**Figure 5:**
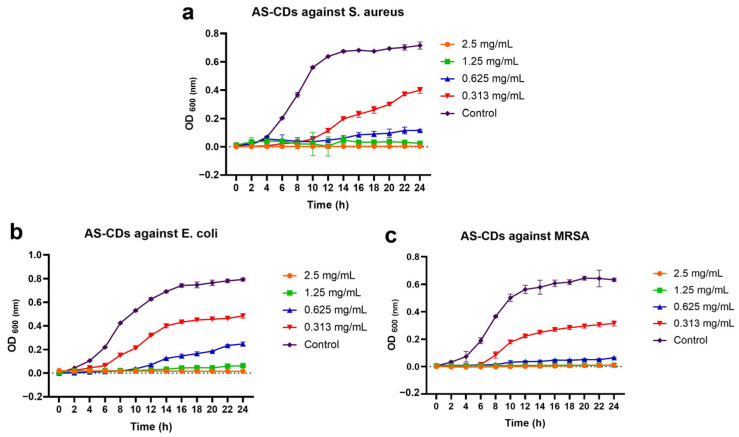
Growth inhibition curves of AS-CDs (0.313–2.5 mg/mL) against *S. aureus* (**a**), *E. coli* (**b**) and MRSA (**c**).

Based on MIC normalization, the tested concentrations corresponded to 1/2×, 1×, 2× and 4× MIC for *S. aureus* and MRSA, and 1/4×, 1/2×, 1× and 2× MIC for *E. coli* (Figure 5a–c). Under these conditions, AS-CDs exhibited a clear dose- and time-dependent inhibition of bacterial growth against all three strains.

For *S. aureus* and MRSA, AS-CDs at ≥2× MIC (1.25 and 2.5 mg/mL) maintained OD_600_ values close to baseline throughout the 24 h incubation, indicating effective and sustained suppression of bacterial proliferation. At 1× MIC (0.625 mg/mL), growth remained strongly inhibited, with no pronounced exponential phase observed. Even at 1/2× MIC (0.313 mg/mL), bacterial growth was noticeably delayed and the final OD_600_ was substantially lower than that of the untreated control, demonstrating a persistent inhibitory effect under sub-MIC exposure. In the case of *E. coli*, strong growth suppression was observed at 1× and 2× MIC (1.25 and 2.5 mg/mL), whereas 1/2× and 1/4× MIC resulted in partial growth recovery. Nevertheless, sub-MICs still led to reduced growth rates and lower terminal OD_600_ values compared with the control, indicating incomplete but measurable inhibition.

The antibacterial effects observed in this study may be associated, at least in part, with interfacial interactions between the AS-CDs and bacterial cells, which could contribute to subsequent membrane disturbance and bacterial inactivation [15,31,32]. Previous studies have examined zeta potential changes before and after the interaction between CDs and bacteria and have used these results as supportive evidence when discussing possible antibacterial mechanisms. For example, Zhang et al. used zeta potential analysis to evaluate the surface charge characteristics of CDs and bacterial cells and suggested that electrostatic attraction may facilitate close association between the positively charged CDs and negatively charged bacterial surfaces [33]. Similarly, Luo et al. reported that zeta potential shifts after co-incubation, when interpreted together with fluorescence, SEM, and live/dead staining data, supported a possible multi-step process involving electrostatic adsorption, membrane penetration, and subsequent cellular damage [13]. In the context of the present work, these published studies provide a reasonable basis for considering interfacial charge-mediated interaction as one possible contributor to antibacterial activity. However, such interpretation should be made with caution, since zeta potential measurements mainly reflect the apparent interfacial charge characteristics of the dispersed system and, on their own, do not constitute direct proof of physical contact, specific binding, or the full antibacterial mechanism. Therefore, in future mechanistic studies, zeta potential analysis may be included as an auxiliary approach to help interpret possible material–bacteria interactions, while more direct validation methods will still be needed to more clearly elucidate the antibacterial mechanism.

### 3.4. Antibacterial Activity of AS-CDs and CDs-Ce6 Conjugate with Photoexcitation

**Figure 6:**
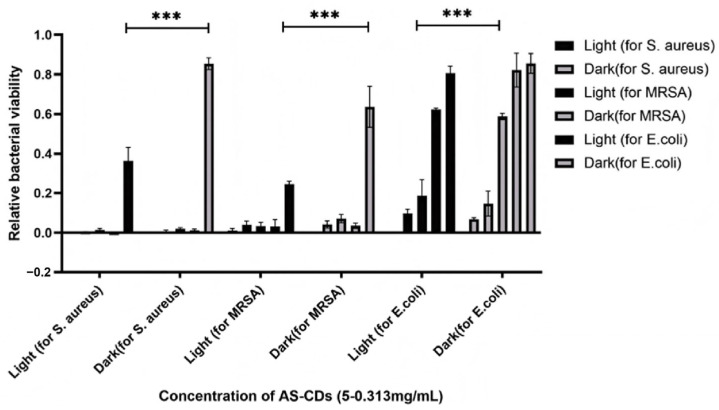
Relative bacterial viability assessed after treatment with AS-CDs (5, 2.5, 1.25, 0.625, 0.313 mg/mL) under light (660 nm, 284 mW/cm^2^, 30 min) and dark conditions. Values were mean (from three replicates) ± standard deviation. One-way ANOVA, *** *p* = 0.001.

The antibacterial effects of AS-CDs and CDs-Ce6 were further evaluated under photoexcitation. As shown in Figure 6, relative bacterial viability was calculated as the ratio of the OD_600_ of bacteria in the treatment group to that of the control group (without AS-CDs and CDs-Ce6 treatment), reflecting bacterial survival. Under light-exposed conditions, AS-CDs demonstrated significantly enhanced antibacterial performance against *S. aureus*, MRSA, and *E. coli* compared to dark conditions (Figure 6).

At a concentration of 5 mg/mL, AS-CDs exhibited excellent bactericidal effects against *S. aureus* and MRSA, with relative bacterial viability approaching zero under both light and dark conditions. However, at a reduced concentration of 0.313 mg/mL, the relative bacterial viability of *S. aureus* and MRSA treated with AS-CDs under light exposure was below 0.4, significantly outperforming the non-light-exposed condition, where viability remained above 0.6.

For *E. coli*, AS-CDs at 5 mg/mL completely eradicated the bacteria under light exposure. At a concentration of 1.25 mg/mL, the relative bacterial viability of *E. coli* dropped below 0.2 under light-exposed conditions, demonstrating significantly enhanced antibacterial activity compared to dark conditions. These results indicated that AS-CDs exhibit superior antibacterial effects under photoexcitation, underscoring the importance of light activation in enhancing their bactericidal activity.

**Figure 7:**
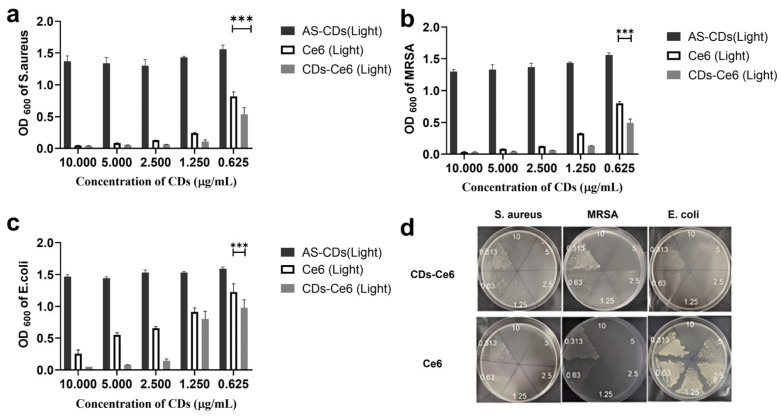
Antibacterial activity of AS-CDs and CDs-Ce6 against *S. aureus*, MRSA, and *E. coli* with and without photoexcitation. (**a**) Comparative antibacterial activity of AS-CDs, Ce6, and CDs-Ce6 against *S. aureus* under photoexcitation. (**b**) Comparative antibacterial activity of AS-CDs, Ce6, and CDs-Ce6 against MRSA under photoexcitation. (**c**) Comparative antibacterial activity of AS-CDs, Ce6, and CDs-Ce6 against *E. coli* under photoexcitation. (**d**) Light-activated antibacterial effects of CDs-Ce6 and Ce6 (0.313–10 μg/mL) on *S. aureus*, MRSA, and *E. coli* after 24 h post-photoexcitation incubation. Values were mean (from three replicates) ± standard deviation. Two-way ANOVA, *** *p* = 0.001.

Under light-exposed conditions, CDs-Ce6 demonstrated significantly enhanced antibacterial activity compared to AS-CDs (Figure 7a–c). Both CDs-Ce6 and Ce6 exhibited notable antibacterial effects at concentrations ranging from 10 μg /mL to 1.25 μg /mL. Within this concentration range, AS-CDs, lacking the photosensitizer Ce6, showed negligible antibacterial activity compared to the control group. At a concentration of 0.625 μg/mL, the absorbance (OD_600_) of *S. aureus* and MRSA treated with CDs-Ce6 under light exposure dropped below 0.5, whereas the absorbance for bacteria treated with Ce6 alone remained above 0.75. This highlighted the superior antibacterial efficacy of the CDs-Ce6 conjugate, driven by the synergistic effect of Ce6 conjugation.

For *E. coli*, CDs-Ce6 under light exposure significantly inhibited bacterial growth across concentrations ranging from 10 μg/mL to 0.625 μg/mL, demonstrating enhanced antibacterial activity compared to AS-CDs. While the antibacterial effect at 0.625 μg/mL was less pronounced against *E. coli* than against *S. aureus* and MRSA, it was still markedly higher than that of Ce6 alone under light exposure. These results confirm that CDs-Ce6 conjugates significantly enhance light-activated antibacterial efficacy compared to Ce6 without conjugation. After 30 min of light irradiation and subsequent 24 h incubation, the antibacterial activity of CDs-Ce6 and free Ce6 was compared against *S. aureus*, MRSA, and *E. coli* on agar plates (Figure 7d). CDs-Ce6 exhibited markedly stronger antibacterial effects across all tested strains, with nearly complete inhibition at lower concentrations (0.313–1.25 μg/mL), whereas Ce6 alone showed only partial growth suppression at comparable doses. This enhanced efficacy of CDs-Ce6 can be attributed to synergistic effects: the CDs facilitate closer interaction with bacterial surfaces, improve local photosensitizer concentration, and contribute to ROS generation under light irradiation. These results clearly demonstrated that conjugation with CDs significantly improved the photodynamic antibacterial performance of Ce6, particularly against resistant strains such as MRSA.

*S. aureus* and MRSA have a thick peptidoglycan layer that is relatively permeable to small molecules and particles. This structural characteristic potentially facilitates the penetration of ROS and the carbon dots themselves, enabling more effective bacterial inactivation upon light activation [34,35,36]. The generated ROS can readily traverse the simpler cell wall structure, causing significant damage to critical cellular components, ultimately leading to cell death.

In contrast, *E. coli* features an outer membrane rich in lipopolysaccharides, which acts as a robust barrier against many substances, including nanoparticles and ROS [35,37]. This outer membrane provides *E. coli* with substantial protection against external oxidative stress, reducing the efficacy of ROS generated by light-activated AS-CDs and CDs-Ce6 conjugates. Even when ROS are generated effectively, the outer membrane of *E. coli* may prevent these reactive species from reaching vital intracellular targets, such as DNA, proteins, and internal membranes, thereby diminishing the bactericidal effect.

This structural difference likely explains why N-doped CDs and CDs-Ce6 conjugates in this study exhibited less effective photobacterial activity against *E. coli* compared to their stronger effects on *S. aureus* and MRSA. These findings underscore the critical role of bacterial structural differences in determining the success of such treatments and highlight the need for tailored therapeutic strategies depending on the target bacterial species.

### 3.5. ROS Generation of CDs and CDs-Ce6 Conjugate

**Figure 8:**
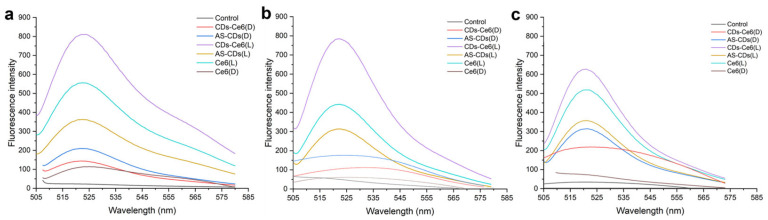
Fluorescence intensity of *S. aureus* (**a**), MRSA (**b**), and *E. coli* (**c**) after treatment with DCFH-DA, showing ROS generation. The data are presented as color-coded lines comparing the effects of AS-CDs (1.25 mg/mL), CDs-Ce6 conjugates (5 μg/mL), and Ce6 (5 μg/mL) under photoexcitation (L) or dark conditions (D).

Bacterial killing can be induced through oxidative stress caused by ROS [38]. Intracellular ROS formation can be quantified by measuring the increase in fluorescence intensity of DCF. DCFH-DA penetrates the bacterial cell membrane and is hydrolyzed by intracellular esterases to non-fluorescent 2,7-dichlorodihydrofluorescein (DCFH). In the presence of ROS, DCFH is oxidized to 2,7-dichlorofluorescein (DCF), a highly fluorescent compound [26]. Fluorescence spectra of treated bacterial samples interacting with DCFH-DA are shown in Figure 8.

Compared to the control group (no treatment), bacterial samples treated with AS-CDs, Ce6, and CDs-Ce6 in the dark exhibited higher fluorescence intensity. This indicates that even in the absence of light exposure, carbon dots, photosensitizers, and their conjugates can generate ROS. This dark-state ROS generation could be attributed to spontaneous electron transfer involving the surface functional groups of the carbon dots (e.g., –OH, –NH_2_, –COOH) and the conjugated Ce6 molecules, which may weakly interact with dissolved oxygen to produce small amounts of ROS [39]. The resulting ROS levels under these conditions are substantially lower than those generated upon light irradiation and are therefore expected to contribute minimally to the antibacterial activity. Such low-level ROS formation in the absence of light has been reported for carbon-based nanomaterials and photosensitizers and is considered a minor, background oxidative process. Under photoexcitation, significantly higher fluorescence intensities were observed in samples treated with AS-CDs, Ce6, and CDs-Ce6, confirming increased ROS production upon light exposure.

ROS exerts bactericidal effects by inducing oxidative stress within bacterial cells, leading to damage of critical cellular components such as lipids, proteins, and DNA [40]. This oxidative damage disrupts bacterial membranes, inactivates metabolic enzymes, and impairs DNA function, resulting in metabolic dysfunction and cell death. Additionally, ROS can overwhelm bacterial antioxidant defense systems, further exacerbate cellular damage and enhance the antibacterial efficacy of CDs and their conjugates [38,41].

Among the tested groups, CDs-Ce6 conjugates demonstrated the highest ROS generation under light exposure, as evidenced by the strongest fluorescence intensity. This superior ROS production corresponded to the most potent antibacterial effects, consistent with the results of previous antibacterial activity evaluations in this study.

### 3.6. Cellular Toxicity of AS-CDs and CDs-Ce6 Conjugate

**Figure 9:**
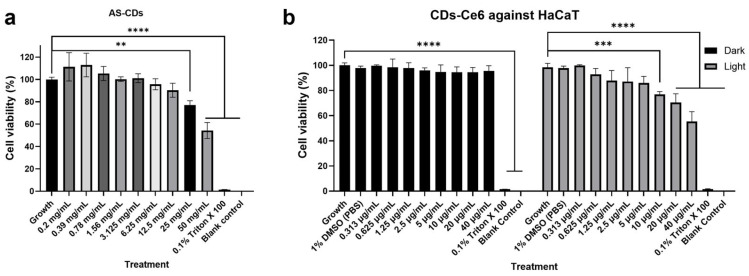
Cytotoxicity evaluation of AS-CDs (**a**) and CDs-Ce6 conjugate (**b**) on HaCaT cells. Error bars represent the standard error of the mean (SEM) from nine replicates of three independent experiments. Statistical analysis was conducted with one-way ANOVA analyses. Error bars represent the mean SEM. ** for *p* ≤ 0.01; *** for *p* ≤ 0.001; **** for *p* ≤ 0.0001.

The cytotoxicity of AS-CDs and CDs-Ce6 conjugates on HaCaT cells was evaluated to assess their biocompatibility for potential biomedical applications. As shown in Figure 9a, AS-CDs exhibited minimal cytotoxicity, with cell viability remaining above 80% after 24 h of incubation even at 20-fold MIC (12.5 mg/mL). This indicated that the AS-CDs synthesized in this study have excellent biocompatibility, making them promising candidates for antimicrobial applications with negligible cytotoxic effects. Figure 8b displayed the cell viability of HaCaT after being treated with CDs-Ce6 conjugates with and without photoexcitation at a concentration range of 0.313 to 40 µg/mL. When treated in the dark, CDs-Ce6 hardly showed cytotoxicity towards HaCaT. After light treatment, CDs-Ce6 showed significant cytotoxicity at concentrations ≥ 10 μg/mL, which were far above its antibacterial working concentration (1.25 μg/mL). These results underscore the suitability of both AS-CDs and CDs-Ce6 conjugates for safe biomedical applications.

## 4. Conclusions

In this study, nitrogen-doped carbon dots (AS-CDs) were synthesized using an advanced microwave-assisted method with L-ascorbic acid and spermidine as precursors. This approach enabled rapid, energy-efficient synthesis of AS-CDs with controlled particle size, uniform morphology, and functional surface groups. The AS-CDs were further functionalized with the photosensitizer Chlorin e6 (Ce6), resulting in the formation of CDs-Ce6 conjugates. Comprehensive characterization using UV-Vis spectroscopy, fluorescence spectroscopy, FT-IR, TEM, DLS, and XRD confirmed the structural integrity and functional properties of both AS-CDs and CDs-Ce6 conjugates. The antibacterial efficacy of AS-CDs and CDs-Ce6 was evaluated against *E. coli*, *S. aureus*, and MRSA. AS-CDs demonstrated significant antibacterial activity, particularly under light exposure, with minimal cytotoxicity to HaCaT cells, suggesting their biocompatibility. The positive zeta potential of AS-CDs facilitated strong interactions with negatively charged bacterial surfaces, enhancing their antibacterial performance. Notably, the CDs-Ce6 conjugates exhibited superior antibacterial activity compared to AS-CDs and standalone Ce6, achieving complete eradication of *S. aureus* and MRSA at low concentrations under light exposure. The enhanced activity of CDs-Ce6 under photoexcitation highlights the synergistic interplay between the carbon dots and Ce6, driven by effective ROS generation.

This study underscores the potential of microwave-synthesized, nitrogen-doped carbon dots as versatile platforms for enhanced photodynamic antibacterial therapy. The superior performance of CDs-Ce6 in overcoming bacterial resistance, particularly under light activation, paves the way for their application in combating antimicrobial resistance and treating bacterial infections more efficiently. However, as the present work is limited to in vitro antibacterial evaluation, further studies are needed to optimize these materials and to validate their efficacy in in vivo wound infection models, which would help to better assess their therapeutic potential and translational value, as well as to explore their broader biomedical applications.

## Data Availability

The original contributions presented in this study are included in the article. Further inquiries can be directed to the corresponding author.
